# Global Associations between Copy Number and Transcript mRNA Microarray Data: An Empirical Study

**DOI:** 10.4137/cin.s342

**Published:** 2008-02-09

**Authors:** Wenjuan Gu, Hyungwon Choi, Debashis Ghosh

**Affiliations:** 1 Department of Biostatistics, University of Michigan, Ann Arbor, MI 48109, U.S.A; 2 Department of Statistics and Huck Institute of Life Sciences, Penn State University, University Park, PA 16802, U.S.A

**Keywords:** circular binary segmentation, high-dimensional data, machine learning, two-color microarray platform

## Abstract

With an increasing number of cancer profiling studies assaying both transcript mRNA and copy number expression levels, a natural question then involves the potential to combine information across the two types of genomic data. In this article, we perform a study to assess the nature of association between the two types of data across several experiments. We report on several interesting findings: 1) global correlation between gene expression and copy number is relatively weak but consistent across studies; 2) there is strong evidence for a cis-dosage effect of copy number on gene expression; 3) segmenting the copy number levels helps to improve correlations.

## Introduction

With the explosion of high-throughput technologies for measuring various aspects of molecular activity, it has become possible to globally monitor the biochemical activities of cells. Much of the application of these technologies has been in the area of cancer, one major example of which is transcript mRNA microarrays (Schena, 2002). There is now a vast literature on microarray studies done in cancer; a simple PubMed search of the phrase “microarray data, cancer” turns up approximately 2400 entries, 99% of which have been published since 2000.

While there have been many examples of individual molecules, genomic signatures and pathways that have been discovered as being dysregulated in cancer through analyses of gene expression data, a more promising avenue is arising based on consideration of integrating genomic data sources in order to validate previous findings based on gene expression studies and to decipher higher-order modular mechanisms of co-expressed genes enriched in various molecular pathways (Rhodes and Chinnaiyan, 2005; Segal et al. 2005).

In cancer, chromosomal aberrations occur frequently. Various types of cytogenetic aberrations, including segmental amplification/deletion and unbalanced translocation events, are a major characteristic of the majority of epithelial tumors. These complex transformations lead to activation of oncogenes and inactivation of tumor suppressor genes. There are many examples of genes that undergo amplifications in cancer, including AKT2 in ovarian cancer, ERRB2 in breast and ovarian cancer, MYCL1 in small cell lung cancer, MYCN in neuroblastoma and EGFR in glioma and non-small cell lung cancer; a comprehensive account of these and other cancer-related genes can be found in Futreal et al. (2004). It has also been shown that many of these changes correlate with clinical factors, such as survival, stage and response to treatment. As an example, for metastatic breast cancer patients with the ERBB2 gene amplification, trastuzumab (Herceptin) is a targeted therapy that is available to achieve a better prognosis.

There are now studies in which microarray data on both an mRNA transcript level as well as a copy number level are being collected. As demonstrated in Virtaneva et al. (2001), a correlation exists between copy number and gene expression in cancer. There are other potential advantages to the integration of copy number and gene expression data. Ludwig and Weinstein (2005) write that the integration of mRNA transcript and copy number information could lead to the identification of new biomarkers. Similarly, Myllykangas and Knuutila (2006) write that integration of copy number and gene expression data can potentially be used to “understand the effects of gene regulation and transcription in amplification manifestation.” With the recently funded Cancer Genome Atlas (http://cancergenome.nih.gov), multiple levels of genomic data are being collected on tumor samples. This will be a valuable resource to the cancer research community. A key question then will be how to combine such genomic datasets.

While there are currently few studies in which both transcript mRNA and copy number microarrays are collected on matched samples, we give two examples which have yielded promising results. In a study by Garraway et al. (2005), the authors examined Affymetrix SNP array data generated on the celebrated NCI-60 cell line data and then sought to find alterations between tissue-specific cancers. Based on such differences, they then looked for differentially expressed genes in the regions that showed overamplification in the corresponding cell lines. These analyses implicated a new candidate oncogene, MITF. In another study using copy number and gene expression study data from the NCI 60 cell line dataset, Bussey et al. (2006) explored the behavior of 64 candidate oncogenes based on correlations between gene expression, copy number and compound activity score. Based on these correlations, they were able to nominate some candidate biomarkers. We term studies of this type as being in the area of cancer “integromics,’’ where data measuring different biochemical activities are measured on the same samples. They demonstrate the potential of such datasets to better identify candidate biomarkers in disease progression and prognosis.

In this study, global associations between gene expression and copy number are examined across studies of multiple cancer types. This analysis was motivated for the following reasons. First, knowing the general pattern of correlation structure between the two data types allows us to assess the feasibility of integrative analysis in terms of expected (within-sample) signal-to-noise ratio and variability of the association between copy number and gene expression across samples. Second, it is important to know if the correlation between gene expression and copy number generalizes across different sites of origin. Third, correlation between the two types of data may differ by the nature of samples. In particular, copy number characterization will be distinct depending on whether the study is *in vivo* or *in vitro*. Much of the results reported will be of a descriptive nature. Finally, we will explore higher-order correlations using modern machine learning methodologies (Efron et al. 2004) as well as bioinformatic methods for segmenting genomic data (Olshen et al. 2004).

## Methods

### Datasets

Only genomic datasets in which the same samples were profiled on a genomic DNA and transcriptomic basis using two-channel microarray platforms were considered here. Use of the same array (probe) designs on both data types avoids having to perform inter-platform identification mapping of genes.

We searched the Stanford microarray database (http://genome-www5.stanford.edu/), Gene Expression Omnibus (http://www.ncbi.nlm.nih.gov/geo/) and ArrayExpress (http://www.ebi.ac.uk/arrayexpress/) in November 2006 for such complete datasets. Datasets from the following published studies were used: Pollack et al. (2002); Hyman et al. (2002); Heidenblad et al. (2005); Zhao et al. (2005) and Kim et al. (2006). Here and in the sequel, we will refer to these datasets by the name of the first author. There are three things we note about the datasets. First, all of the datasets except for that from Pollack et al. (2002) are from cancer cell lines. Second, the cell lines are from different tissues of origin. This will allow us to observe tissue-specific phenomena. Third, the Hyman et al. (2002) and Pollack et al. (2002) breast datasets will allow us to assess concordance of copy number/transcript correlations between *in vivo* and *in vitro* systems.

## Data Preprocessing

Missing data were imputed using a k-nearest neighbors algorithm with k = 5 (Troyanskaya et al. 2001). Then, a variance filter was applied to the transcript mRNA and copy number micro-array datasets separately. The spots with the lowest 25% variance across all samples were excluded from each dataset. This was done to ensure that the correlations calculated in the study would not be subject to artefacts arising from experimental noise. The final numbers of genes and samples from the available studies are listed in Table 1.

## Results

### Univariate correlation study

As an initial analysis, we calculated Pearson correlation between the expression level of every gene with its corresponding copy number expression profile. This is shown in Table 2.

Comparing the median values for correlation between transcript mRNA and copy number across the five studies is equivalent to the copy number explaining 12%–40% of the variation in gene expression across the five studies. Compared to the 12% figure given by Pollack et al. (2002), we find evidence of stronger association in the cell line datasets than in the human tissue. In general, we find weak correlation globally between the transcript mRNA and copy number expression levels. However, on average, we can also say that the correlation distribution appears fairly consistent across studies, with the one exception being the Zhao et al. study, which appears to have a greater amount of variability than the other studies. This is probably due to the small sample size of the study; it has the lowest number of samples among the studies.

We next summarized all possible pairwise univariate correlations between gene expression and copy number. The results are given in Table 3.The correlation between a gene’s expression level and corresponding copy number rarely represents the largest correlation in terms of magnitude in the data-set. Table 3 suggests that there tends to be stronger correlation between transcript levels and copy number if we consider genes from the same chromosome; however, the increase in the proportion of genes with high correlation remains modest. This is a consistent finding with the notion of regulation of gene expression not being a simple relationship. It is also suggestive of a so-called “cis-dosage’’ effect of copy number on transcription. We further explored this association using more modern data mining techniques in the next section.

### Higher-order correlation studies

While Table 1 provides a nice summary of the univariate correlations, we also explored higher-order correlation modelling strategies. In particular, we wished to allow for the effects of copy number expression from multiple loci to influence the transcription level of a gene.

The approach we tried was *Least Angle Regression* (“LARS”) (Efron et al. 2004). It involves fitting a linear regression model in which the response variable is gene expression and the copy number expression values from all loci are potential predictors. Since the possible number of predictors is much greater than the number of samples, it is impossible to obtain a unique estimate of the linear regression model. The LARS algorithm allows the user to fit such a model; its estimation procedure involves a sequential and iterative fitting procedure where only m sequential steps are taken. In our case, we take *m* = (number of samples)/2. This implies that a large fraction of the regression coefficients will be estimated to be exactly zero. Below is a description of the LARS procedure:

1. All coefficients are equal to zero at the beginning, find the predictor most correlated with the response, denote as *x_j1_*.
2. With *x_j1_* we find *x_j2_* which is most correlated with the current residual vector.
3. Proceed equiangularly between the two predictors until a third covariate *x_j3_* enters the “most correlated” set.
4. Proceed equiangularly between *x_j1,_* *x_j21_* and *x_j3_* until the fourth covariate enters, and so on.

After *m* steps, we stop. Thus in the final results of the regression, for each response there are only *m* covariates have non-zero estimated coefficients. Such an approach allows for multiple copy number expression values to influence a given gene’s mRNA transcript levels.

Given the results of the LARS analyses for each gene, we can test for a cis-dosage effect. If such an effect exists, then this means that the mRNA expression of one gene is more correlated with the DNA copy number of genes located on the same chromosome than with DNA copy number of genes from different chromosomes. We tested for the presence of a cis-dosage effect using a Wilcoxon test. These results are summarized in Supplementary Table 1.

We draw several conclusions from the table. First, the strength of the cis-dosage effect varies across cancer type. For the purposes of interpreting the table, if we assumed that there was no cis-dosage effect globally, we still would expect approximately one chromosome to show significant evidence of a cis-dosage effect if we are testing at a significance level of 0.05. It is most significant for breast cancer, showing twenty significant chromosomes for the Pollack et al. data and 16 significant chromosomes for the Hyman et al. (2002) data. However, for the prostate cancer cell line there only exist 9 significant chromosomes. The correlation patterns for cancer tumor tissue and cell lines from the Pollack and Hyman et al. data, which are both from breast cancer, are similar in that both show strong overall location effect. In conclusion, based on our analysis, the relationship between copy number and expression level is appears to be greater than captured by simple correlation, and the significance of this relationship varies for different cancer types.

The other correlation analysis we performed was to take into account that copy number and gene expression levels might have spatial correlation. This is accommodated through the use of segmentation techniques (Olshen et al. 2004). In particular, we repeated the correlation of analysis in Table 2 by segmenting both the copy number and the gene expression data. Various correlations were calculated and are summarized in Table 4. This table shows that the single gene copy number-transcript mRNA correlations improve by incorporating segmentation methods into the analysis. This also suggests that incorporating the spatial correlation in gene expression and copy number expression will improve the amount of gene expression variation that is explained by copy number expression.

## Discussion

In this study, we have explored the nature of correlation between transcript mRNA and copy number for genes across five cancer profiling studies in which both types of measurements were collected on the same samples. Three findings are notable from this study:

1. The global nature of correlation between transcript mRNA and copy number is in accordance with what is reported in Pollack et al. (2002) and appear to be consistent across studies.
2. There is solid evidence for a cis-dosage effect of copy number on transcription.
3. Segmentation of genomic data leads to better correlation, even more than what has been suggested by Pollack et al.

The first result suggests that other phenomena might control and hence be able to explain the variation in gene expression. Examples include histone modifications, mutations in DNA sequence, microRNA molecules and protein activity. This study outlines that there are still many factors that are needed to explain gene expression. With the explosion in new technologies, the effects of other factors on gene expression could potentially be studied. A second and more statistical point is that the weak overall correlation suggests that the approach authors have taken of focusing on specific genes or molecules from “integromic’’ analyses of gene expression and copy number data seem to be quite reasonable. If there were stronger correlations between gene expression and copy number, then there would be potential advantage in pooling results from spatially contiguous genes; however, this approach might not lead to any gains in power, given the nature of correlation that we are finding here. Of course, if there are sample-specific artefacts, then using the correlation coefficient across samples might lead to erroneous conclusions.

One major limitation of our study is that we are not considering clinically heterogeneous samples at all, i.e. we treat each study as having samples coming from one population. With samples from the Cancer Genome Atlas, there will be linked clinical information to the genomic data. It might be the case that the nature of correlation will change depending on certain clinical parameters. This was the approach implicitly utilized by Bussey et al. (2006) and is potentially powerful for finding new biomarkers. How to incorporate clinical information into the analysis is an open area of research and is currently under investigation.

Much of the results we have reported have been based on descriptive measures. A challenging issue is how to perform statistical inference. While permutation procedures are quite popular, it is not clear what should be permuted. In addition, if one wishes to incorporate spatial dependence, then clearly a model-based approach is needed.

Finally, this should be viewed as an initial study in the area. With the possibility of larger-sized datasets emerging in the future, the nature of copy number and gene expression will be more reliably assessed. By finding consistent patterns across the individual datasets, this provides some strength of evidence to our study.

## Supplementary Material

**Table S1.** Results of the LARS analyses to find cis-dosage effects of copy number on gene expression. The results are grouped by first study author. Chrm refers to chromosome. Average number of non-zero betas denotes the average number of copy number expression measurements from the same chromosome found to have a non-zero effect on gene expression for all genes on the chromosome. Expected number of non-zero betas denotes the average number of non-zero betas under the null hypothesis of no-cis dosage effect. P-value is based on the Wilcoxon rank-sum test.

## Figures and Tables

**Table 1:** Description of datasets used in the analysis. Dataset column refers to first author on publication containing data.

Dataset	Organ site	In vivo/in vitro	Number of genes	Number of samples
Pollack	Breast	In vivo	4841	36
Hyman	Breast	In vitro	6823	14
Heidenblad	Pancreas	In vitro	8879	16
Zhao	Prostate	In vitro	14824	8
Kim	Lung	In vitro	21066	22

**Table 2:** Summary statistics for pair wise correlation between copy number and expression.

Dataset	Min	1st Quantile	Median	Mean	3rd Quantile	Max	SD
Pollack	−0.49	−0.02	0.11	0.12	0.26	0.90	0.22
Hyman	−0.83	−0.07	0.14	0.15	0.36	0.99	0.31
Heidenblad	−0.79	−0.04	0.16	0.15	0.34	0.92	0.28
Zhao	−0.95	−0.14	0.19	0.16	0.48	0.99	0.41
Kim	−0.79	0.02	0.22	0.20	0.39	0.94	0.27

**Notes:** Min refers to minimum; Max refers to maximum. SD is standard deviation.

**Table 3:** Summary of univariate correlation results across five studies.

Dataset	Number of genes	# genes most correlated with themselves	# genes most correlated with genes from the same chromosome
Pollack	4841	42 (0.90%)	619 (13.21%)
Hyman	6823	28 (0.41%)	648 (9.50%)
Heidenblad	8879	8 (0.09%)	713 (8.03)
Zhao	14284	3 (0.02%)	853 (6.09%)
Kim	21066	79 (0.5%)	2344 (14.0%)

**Notes:** In this table, the second column refers to the number of genes in which the largest correlation in magnitude with copy number is that from the same gene (i.e. the same spot on the microarray). The third column refers to the number of genes whose largest correlation between expression and copy number was with a spot that mapped to a gene on the same chromosome.

**Table 4:** Correlations across five datasets taking segmentation into account.

**Hyman**	**0%**	**25%**	**50%**	**75%**	**100%**	**SD**	**MAD**
Both Unsegmented	−0.859	−0.057	0.159	0.373	0.956	0.303	0.246
Expression Segmented	−0.805	−0.025	0.211	0.432	0.974	0.317	0.259
Copynumber Segmented	−0.902	−0.022	0.219	0.453	0.969	0.324	0.267
Both Segmented	−0.734	0.259	0.471	0.652	0.976	0.281	0.224
**Heidenblad**	**0%**	**25%**	**50%**	**75%**	**100%**	**SD**	**MAD**
Both Unsegmented	−0.777	−0.051	0.147	0.34	0.949	0.276	0.224
Expression Segmented	−0.746	0.069	0.268	0.463	0.948	0.285	0.229
Copynumber Segmented	−0.804	−0.008	0.196	0.395	0.952	0.288	0.234
Both Segmented	−0.691	0.247	0.464	0.645	0.975	0.288	0.231
**Pollack**	**0%**	**25%**	**50%**	**75%**	**100%**	**SD**	**MAD**
Both Unsegmented	−0.547	−0.028	0.106	0.248	0.898	0.208	0.165
Expression Segmented	−0.607	0.082	0.241	0.399	0.947	0.226	0.183
Copynumber Segmented	−0.54	0.005	0.166	0.331	0.914	0.231	0.186
Both Segmented	−0.396	0.327	0.479	0.62	0.971	0.214	0.172
**Zhao**	**0%**	**25%**	**50%**	**75%**	**100%**	**SD**	**MAD**
Both Unsegmented	−0.946	−0.127	0.196	0.488	0.989	0.406	0.338
Expression Segmented	−0.939	0.085	0.42	0.675	0.99	0.401	0.331
Copynumber Segmented	−0.968	−0.115	0.227	0.525	0.99	0.418	0.348
Both Segmented	−0.923	0.309	0.623	0.819	0.997	0.383	0.304
**Kim**	**0%**	**25%**	**50%**	**75%**	**100%**	**SD**	**MAD**
Both Unsegmented	−0.803	0.051	0.241	0.42	0.972	0.263	0.214
Expression Segmented	−0.726	0.124	0.328	0.522	0.932	0.279	0.228
Copynumber Segmented	−0.722	0.077	0.287	0.479	0.957	0.278	0.228
Both Segmented	−0.644	0.275	0.482	0.675	0.924	0.282	0.228

**Notes:** SD represents standard deviation; MAD represents mean absolute deviation. Both Unsegmented refers to correlation coefficients between gene expression and copy number based on the raw expression number value, after taking the preprocessing steps described in the paper. Expression Segmented refers to running the algorithm of Olshen et al. (2004) on the gene expression data on individual samples and to then calculate the correlation coefficients between gene expression and copy number expression. Copynumber Segmented refers to running the algorithm of Olshen et al. (2004) on the copy number expression data on individual samples and to then calculate the correlation coefficients between gene expression and copy number expression. Both Segmented refers to running the algorithm of Olshen et al. (2004) on the copy number and gene expression data on individual samples and to then calculate the correlation coefficients between gene expression and copy number expression.
